# Canonical microRNA loss drives tumor development, implicating therapeutic efficacy of enoxacin in angiosarcoma

**DOI:** 10.1261/rna.080706.125

**Published:** 2026-06

**Authors:** Bozhi Liu, Ant Murphy, Annaleigh Benton, Lauren Gartenhaus, William Budka, Juliana M. Bronzini, Samuel Hartzler, Madison E. Yates, Alejandra Romero Alzate, Nimod D. Janson, Shyaman Jayasundara, Sagar Utturkar, Nadia A. Lanman, Majid Kazemian, Jason A. Hanna

**Affiliations:** 1 Department of Biological Sciences, Purdue University, West Lafayette, Indiana 47906, USA; 2 Purdue Institute for Cancer Research, Purdue University, West Lafayette, Indiana 47906, USA; 3 Department of Biochemistry and Computer Science, Purdue University, West Lafayette, Indiana 47906, USA; 4 Department of Comparative Pathobiology, Purdue University, West Lafayette, Indiana 47906, USA

**Keywords:** angiosarcoma, enoxacin, miRNA, sarcoma

## Abstract

Angiosarcoma (AS) is a rare and aggressive tumor arising within the endothelium, characterized by a high metastatic rate and poor prognosis. Our prior work established that endothelial loss of *Dicer1*, a key enzyme in microRNA (miRNA) processing, drives AS formation in mice, indicating a tumor suppressive role for miRNAs in tumorigenesis. Here, we corroborated this hypothesis by generating a novel conditional knockout model targeting *Dgcr8*, a core component of the microprocessor complex required for pri-miRNA processing. Conditional deletion of *Dgcr8* phenocopies *Dicer1* loss, resulting in spontaneous AS formation and global loss of mature miRNAs. We further demonstrate that treatment with enoxacin (ENX), a repurposed antibiotic known to enhance miRNA processing, reduces viability, migration, and clonogenicity of AS cells. ENX increases the abundance of tumor-suppressive miRNAs and downregulates oncogenic pathways, including pathways related to cell cycle progression, angiogenesis, and cell migration. These results establish the essential role of miRNA biogenesis in suppressing AS and reveal a pharmacologically targetable vulnerability via ENX-mediated enhancement of miRNA expression in tumors.

## INTRODUCTION

Angiosarcoma (AS) is a rare tumor arising within the vascular or lymphatic endothelium and can occur in virtually any anatomic location. Comprising 2% of all soft tissue sarcomas in humans, AS is notoriously malignant with a metastatic rate of 44% and a 5 year overall survival of 20%–30% (Cao et al. 2019; Florou and Wilky 2021; Wagner et al. 2024). The molecular mechanisms underlying AS initiation and progression are not well understood; however, varied risk factors for AS have been identified, including chronic lymphoedema, therapeutic radiation, UV exposure, exposure to carcinogenic compounds such as vinyl chloride, and familial genetic syndromes (Antonescu 2014; Benton et al. 2024a). Treatments for AS are varied: Surgery, radiotherapy, and chemotherapy are utilized depending on anatomic site, size, and presence of metastasis (Cao et al. 2019). The diversity of AS necessitates the need for new models and tools to help understand the diverse mechanisms driving AS progression and how to address them pharmacologically.

We previously demonstrated that conditional deletion of *Dicer1* in endothelial cells and subsequent loss of mature microRNAs (miRNAs) leads to angiosarcoma development in a genetically engineered mouse model (Hanna et al. 2017). miRNAs are regulatory RNAs, which attenuate gene expression by either promoting degradation of specific mRNA transcripts, or by repressing their translation (Shang et al. 2023). miRNAs are important regulators in every cell type and in virtually every cellular process (Bartel 2018). They are transcribed by RNA Pol II as long transcripts, referred to as primary miRNAs (pri-miRNAs), which are then trimmed to smaller hairpins (pre-miRNAs) in the nucleus by the Microprocessor complex, consisting of DROSHA and DGCR8 (DiGeorge critical region 8) enzymes(Lin and Gregory 2015). These RNA hairpins are exported to the cytoplasm via Exportin V where they are then processed to the final mature miRNA by DICER1 (Kim et al. 2025). The mature miRNAs are then loaded into RNA-induced silencing complexes (RISCs) containing the Argonaut (Ago) family of proteins. The miRNA loaded RISC complex is then directed to target mRNAs based on partial miRNA–mRNA complementarity (Denzler et al. 2016). This interaction leads to mRNA degradation and translational repression, ultimately downregulating the expression of the regulated genes.

Dysfunction of DGCR8 is well-known to be associated with DiGeorge syndrome, a rare genetic disorder in which a segment of Chromosome 22 (including *DGCR8*) is deleted, causing a diverse set of symptoms including learning disabilities, congenital heart disease, weakened immune system, scoliosis, among others (Shiohama et al. 2003). In addition to the wide range of disorders caused by loss of *DGCR8*, mutations and loss of heterozygosity of *DGCR8* have been associated with cancer (Rivera et al. 2020; Paulsson et al. 2021). Driver mutations in both microprocessor genes, *DGCR8* and *DROSHA*, have been identified in Wilms tumor and this leads to dysregulated miRNA processing (Rakheja et al. 2014; Vardapour et al. 2022; Tiburcio et al. 2024).

Enoxacin (ENX), a fluoroquinolone antibiotic, has been discovered to enhance miRNA processing (Shan et al. 2008; Zhang et al. 2008). Although the precise mechanism is currently unclear, this effect is hypothesized to be accomplished through increasing the affinity between TRBP and pre-miRNAs, leading to enhanced processing by DICER1 (Melo et al. 2011). ENX promotes miRNA activity even in cancer cells with DICER1 downregulation, suggesting a potential therapeutic value by restoring miRNA activity in a biogenesis-impaired context (Ramírez-Moya et al. 2019). Interestingly, studies that have evaluated miRNA abundance in ENX-treated cells indicate a similar number of decreased and increased miRNAs, yet ENX globally represses tumorigenesis and curiously tends to promote suppressive miRNA abundance (Jałbrzykowska et al. 2022).

In our previous work, we demonstrated that conditional loss of *Dicer1* is sufficient to promote AS development using Cre recombinase under the control of the *adipocyte protein 2 (aP2)* promoter (Tang et al. 2008). DICER1 has been implicated in several RNAi-independent functions including regulation of dsRNA sensing and interferon responses (Baldaccini et al. 2024), modulation of chromatin structure and transcription (Reyes-Castro et al. 2023), and roles in genome stability and DNA damage response (Francia et al. 2012). To determine if this tumor phenotype is dependent on canonical miRNA loss and not due to other functions of DICER1, we have conditionally deleted *Dgcr8*, which processes miRNAs upstream of DICER1. We also evaluate the efficacy of ENX and its impact on gene and miRNA expression in AS cells.

## RESULTS

### *aP2-Cre;Dgcr8*^*Flox/Flox*^ mice are viable

**FIGURE 1.**
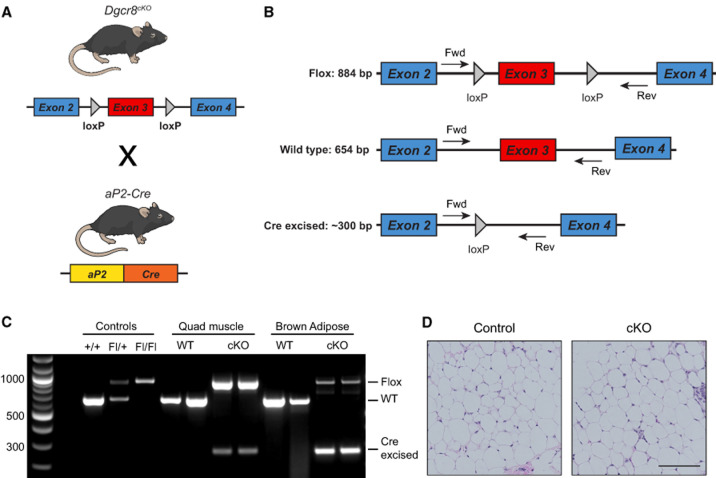
*Dgcr8* conditional deletion with *aP2-Cre*. (*A*) Graphic of *Dgcr8*^*cKO*^ and *aP2-Cre* alleles and breeding scheme to generate *aP2-Cre;Dgcr8*^*cKO*^ animals. Mouse illustration from the NIAID NIH BIOART Source (bioart.niaid.nih.gov/bioart/279). (*B*) Map of primers for genotyping the Flox, wild-type, and Cre excised alleles by genomic PCR. (*C*) Genomic PCR of DNA from controls, quadriceps muscle, or brown adipose tissue of wild-type (WT) or *aP2-Cre;Dgcr8*^*Fl/Fl*^ (cKO) animals. (*D*) Normal inguinal white adipose tissue H&E from a wild-type control (Cre−, Dgcr8^Fl/Fl^) or Cre+, *aP2-Cre;Dgcr8*^*Fl/Fl*^ (cKO) animal; scale bar, 100 µm.

Following our recent observations that conditional *Dicer1* deletion in mice drives AS development (Hanna et al. 2017; Hanna 2022), we sought to determine if global miRNA loss was the driving force behind tumorigenesis or if noncanonical functions of DICER1 could be contributing. Therefore, we tested if the conditional deletion of *Dgcr8* phenocopied *Dicer1* loss and led to AS. We generated *aP2-Cre;Dgcr8*^*Flox/Flox*^ (AD8^cKO^) using the *aP2-Cre* mouse generated by Tang et al. (2008) and the Dgcr8^Flox/Flox^ mouse generated by Rao et al. (2009) (Fig. 1A). Recombination of *Dgcr8* by Cre is accomplished by loxP sites, which flank exon 3, resulting in a nonfunctional transcript (Fig. 1B). *aP2-Cre* expression and *Dgcr8* recombination are observed in adipose tissues as expected with high efficiency but with some retention of the Flox allele, as previously observed (Fig. 1C; Hanna et al. 2017). Despite depletion of *Dgcr8* in adipocytes and endothelial cells, the AD8^cKO^ mice were viable and fertile with no apparent phenotypes or histologic findings to note in any adipose tissue including the inguinal white adipocyte fat pad from adult (8 week old) control and Dcgr8^cKO^ mice (Fig. 1D).

### Biallelic loss of *Dgcr8* drives angiosarcoma

**FIGURE 2.**
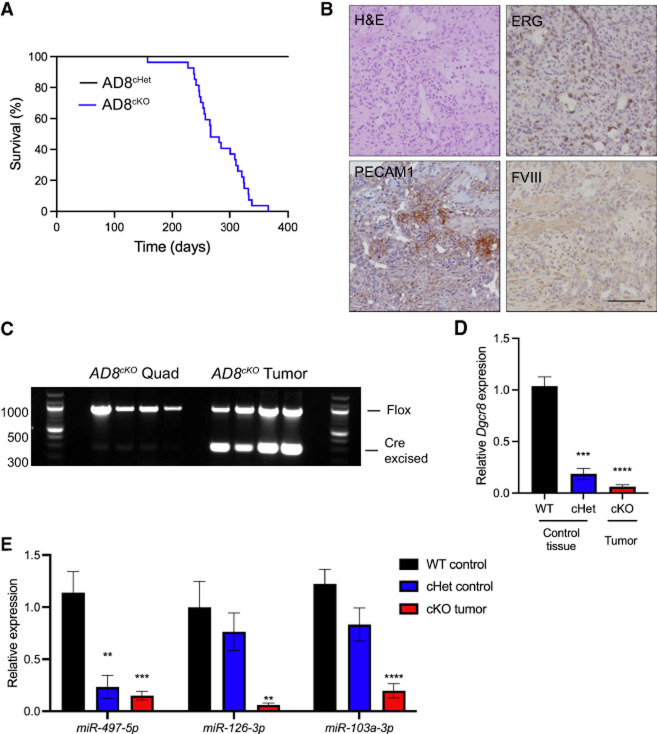
*Dgcr8* deletion leads to angiosarcoma development. (*A*) Kaplan–Meier tumor-free survival curve for *aP2-Cre;Dgcr8*^*cHet*^ (AD8^cHet^, black, *n* = 28) or *aP2-Cre;Dgcr8*^*cKO*^ (AD8^cKO^, blue, *n* = 27, median tumor-free survival time of 266 days); Mantel–Cox log-rank *P* < 0.0001. (*B*) H&E and IHC for ERG, PECAM1, or Factor VIII (FVIII) from an AD8^cKO^ tumor; scale bar, 100 µm. (*C*) Genomic PCR of DNA from AD8^cKO^ control quadricep (quad) muscle or AD8^cKO^ angiosarcomas (*n* = 4). (*D*) Relative expression of *Dgcr8* by RT-PCR from WT and cHet control tissue (*n* = 3) compared to cKO (AD8^cKO^) angiosarcomas (*n* = 3). (*E*) Relative expression of indicated miRNAs by qRT-PCR from tissue as in *D*, (**) *P* < 0.01, (***) *P* < 0.001, (****) *P* < 0.0001.

Loss of a single *Dgcr8* allele (AD8^cHet^) is insufficient for tumorigenesis; however, *aP2-Cre;Dgcr8*^*Flox/Flox*^ (*AD8*^*cKO*^) mice develop AS with a median tumor-free survival of 266 days (Fig. 2A). AS development was 100% penetrant and specific, with hemorrhagic AS tumors being the only tumors that were detected. A panel of endothelial-specific immunohistochemical staining for PECAM1, ERG, and Factor VIII demonstrates the endothelial nature of these tumors, consistent with AS (Fig. 2B). Tumors exhibit a high degree of recombination of *Dgcr8* with the nonrecombined Flox allele, likely amplified from contaminating tumor stroma and normal blood cells (Fig. 2C,D). As expected, *AD8*^*cKO*^ tumors have a significant reduction of *Dgcr8* transcript and mature miRNAs, reflecting the loss of a functional microprocessor complex (Fig. 2D,E). Overall, biallelic loss of *Dgcr8* recapitulates the *Dicer1* deletion phenotype, suggesting that loss of canonical miRNA processing drives tumorigenesis and miRNAs are critical tumor suppressors in AS.

### Enoxacin treatment of AS cells reduces viability, clonogenic potential, and migratory ability

**FIGURE 3.**
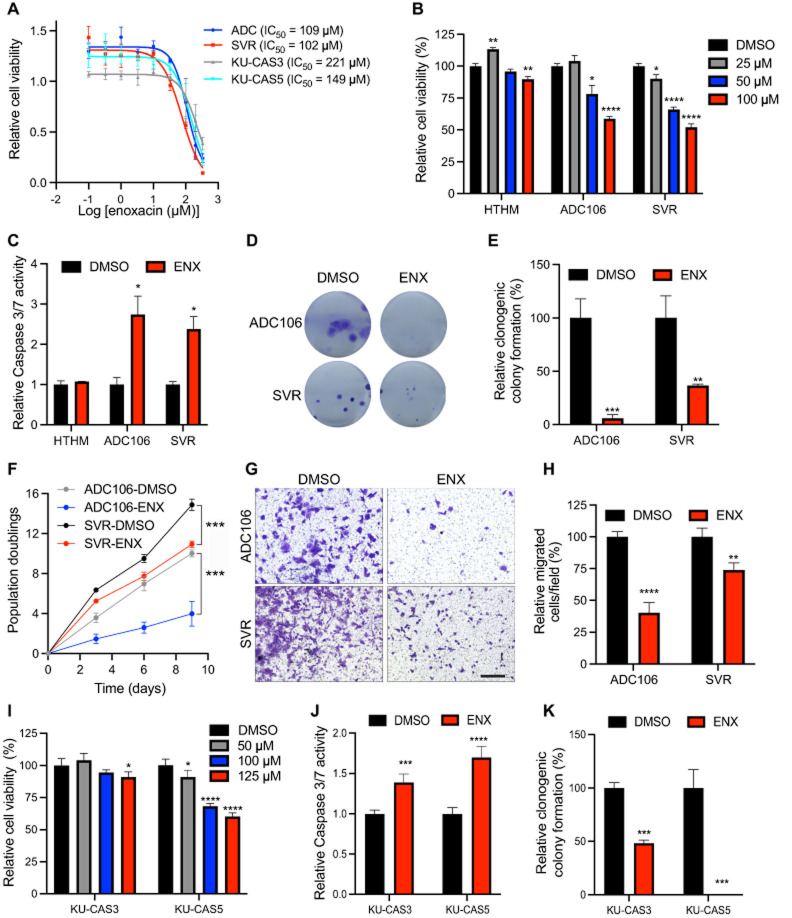
Enoxacin reduces angiosarcoma proliferation. (*A*) Cell viability curve based on CellTiter-Glo analysis of ADC106, SVR, KU-CAS3, and KU-CAS5 cells, 72 h after treatment with indicated concentrations of enoxacin (ENX). (*B*) Relative cell viability via CellTiter-Glo in HTHM, ADC106, and SVR cells treated with the indicated dose of ENX for 72 h. (*C*) Relative Caspase 3/7 activity in ADC106, SVR, and HTHM cells treated with DMSO or 100 µM ENX for 48 h. (*D*) Representative images and (*E*) quantification of clonogenic colony formation in cells treated with DMSO or 100 µM ENX. (*F*) Population doubling assays in ADC106 and SVR cells treated with DMSO or 100 µM ENX. (*G*) Representative image and (*H*) quantification of migrated cells in transwell migration assays in cells treated with DMSO or 100 µM ENX; scale bar, 150 µm. (*I*) Relative cell viability from CellTiter-Glo analysis of human AS cells treated with the indicated concentration of ENX for 72 h. (*J*) Relative Caspase 3/7 activity in cells treated with 125 µM ENX or DMSO for 48 h. (*K*) Quantification of clonogenic colony formation assays in cells treated with DMSO or 125 µM ENX, (*) *P* < 0.05, (**) *P* < 0.01, (***) *P* < 0.001, (****) *P* < 0.0001.

While most cells cannot survive miRNA loss, *Dgcr8* or *Dicer1* deletion in the aP2-Cre model promotes transformation and tumorigenesis, suggesting endothelial cells are uniquely susceptible to miRNA-loss-mediated transformation. Thus, we hypothesized that the restoration of miRNA expression may be particularly efficacious in AS and tested the efficacy of ENX, a known enhancer of miRNA processing and activity in mouse and human AS cell lines including the ADC106, SVR, KU-CAS3, and KU-CAS5. The SVR cell line is an SV40 large T antigen immortalized, HRAS transformed murine AS line, and ADC106 is a cell line derived from an AS tumor that developed in an *aP2-Cre;Dicer1^cKO^;Cdkn2a*^*cKO*^ tumor that retains one Flox allele of *Dicer1* and is therefore functionally heterozygous for *Dicer1* (Arbiser et al. 1997; Hanna 2022). KU-CAS3 and KU-CAS5 cells are human AS cells derived from cutaneous AS of the scalp (You et al. 2022). IC_50_ values in four AS cell lines ranged from 100 to 220 µM (Fig. 3A). Treatment of murine AS cell lines with 100 µM ENX resulted in a significant reduction in cell viability and a significant increase in Caspase 3/7 activity, indicating an increase in apoptosis (Fig. 3B,C). This effect was more pronounced in AS cells since ENX only subtly reduced normal HTHM (HUVEC cells immortalized with hTERT and MYC [Searcy et al. 2023]) but did not induce apoptosis (Fig. 3B,C). ENX-treated AS cells exhibited reduced clonogenic colony formation, population doubling, and migration ability (Fig. 3D–H). Importantly, ENX was still effective in the ADC106 cells with downregulated DICER1 as observed in previous studies (Ramírez-Moya et al. 2019). Similar effects were observed in the human AS cells with ENX treatment, reducing cell viability, increasing apoptosis, and repressing clonogenic colony formation (Fig. 3I–K; Supplemental Fig. S1A).

### Enoxacin treatment leads to increased miRNA expression and transcriptional changes

To determine whether ENX enhances miRNA biogenesis as hypothesized, we first tested its effects using a miR-497 luciferase reporter assay. This reporter, which we previously generated, contains a perfect miR-497 site inserted into the 3′UTR of *Renilla* luciferase (Benton et al. 2024b). The construct was transiently transfected into HEK-293T cells, which were subsequently treated with ENX or DMSO control. As expected, a significant reduction in luciferase activity demonstrated that ENX likely promotes the processing and/or abundance of miR-497 (Supplemental Fig. S1B). Next, the ENX effects on DICER1 and TRBP2 expression were evaluated by immunoblot and qRT-PCR (Supplemental Fig. S1C–H). Subtle increases in *DICER1* mRNA expression were observed in the SVR, KU-CAS3, and KU-CAS5 cells along with increased expression of *TARBP2* mRNA in SVR and KU-CAS5 cells. However, these subtle increases in transcript did not lead to significant changes in protein expression (Supplemental Fig. S1D,E,G,H).

**FIGURE 4.**
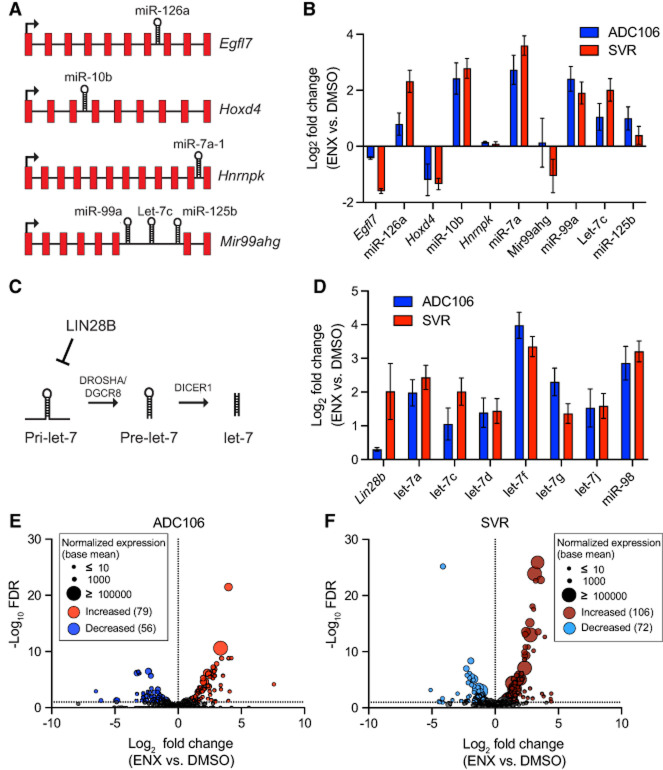
Enoxacin enhances miRNA biogenesis. (*A*) Graphic of intronic miRNAs and their host genes. (*B*) Log_2_ fold change in expression comparing enoxacin (ENX) versus DMSO treated ADC106 (blue) or SVR (red) of intronic miRNAs and host gene transcripts by RNA-seq. (*C*) Graphic of LIN28B regulation of let-7 miRNAs. (*D*) Log_2_ fold change in expression of *Lin28b* and let-7 family miRNAs by RNA-seq. (*E*) Volcano plot of the −log_10_ FDR versus the log_2_ fold change in miRNA abundance with point size representing mean normalized expression (base mean) of ADC106 cells or (*F*) SVR cells treated with DMSO or 100 µM ENX for 72 h. Differentially expressed miRNAs with increased or decreased abundance with FDR < 0.05 and log_2_ fold change >1 and <−1 as indicated.

To determine global effects on miRNA abundance and mRNA expression, we conducted small RNA-seq and mRNA-seq on SVR and ADC106 cells treated with DMSO or ENX for 72 h. To evaluate whether ENX alters miRNA processing rather than transcription, we first examined the expression of several intronic miRNAs and their host genes (Fig. 4A). Increased miRNA abundance was observed without corresponding increases in host gene transcript levels, implicating increased miRNA biogenesis as the driver of miRNA upregulation, not transcription (Fig. 4B). We also evaluated other posttranscriptional mechanisms such as LIN28B regulation of the let-7 family of miRNAs. LIN28B represses the biogenesis of let-7 by inhibiting its processing by DROSHA/DGCR8 in the nucleus (Fig. 4C; Heo et al. 2008; Newman et al. 2008; Viswanathan et al. 2008). Therefore, decreased *Lin28b* expression could increase let-7 abundance (Piskounova et al. 2011). We observed no decrease in *Lin28b* expression, while many let-7 family miRNAs were significantly increased in abundance (Fig. 4D). These findings suggest that let-7 upregulation is not mediated by *Lin28b* downregulation, but rather by other posttranscriptional mechanisms, consistent with the hypothesis that ENX enhances miRNA processing.

We observed 135 differentially expressed miRNAs (absolute log_2_ fold change > 0.5, *P*-value < 0.05, of 499 total miRNAs detected) in ADC106 cells treated with ENX compared to DMSO-treated control cells, with a slight majority being upregulated (79 increased vs. 56 decreased) (Fig. 4E; Supplemental Fig. S2A; Supplemental Table S1). Similarly, ENX-treated SVR cells had 178 miRNAs differentially expressed (106 increased vs. 72 decreased of 642 total miRNAs detected) (Fig. 4F; Supplemental Fig. S2B; Supplemental Table S2). Notably, SVR and ADC106 cells exhibited distinct but overlapping miRNA expression profiles (Supplemental Figs. S2C–E, S3A), with many differentially expressed miRNAs shared between the two cell lines. Notably, the 50 commonly upregulated miRNAs include several known tumor suppressors, such as let-7 family, miR-206, miR-181, miR-146a, miR-335, and miR-126. Additionally, members of the miR-15/16/497/195/424 family, which we have previously shown to be potent tumor-suppressing miRNAs (Benton et al. 2024b), were also increased in the ADC106 cells. We next evaluated the processing of three of these miRNAs (let-7a, miR-126, and miR-146a) to determine if ENX resulted in decreased abundance of the pre-miRNA and increased mature miRNA. Indeed, increased ENX treatment results in increased mature and decreased pre-miRNA (Supplemental Fig. S3B–E).

**FIGURE 5.**
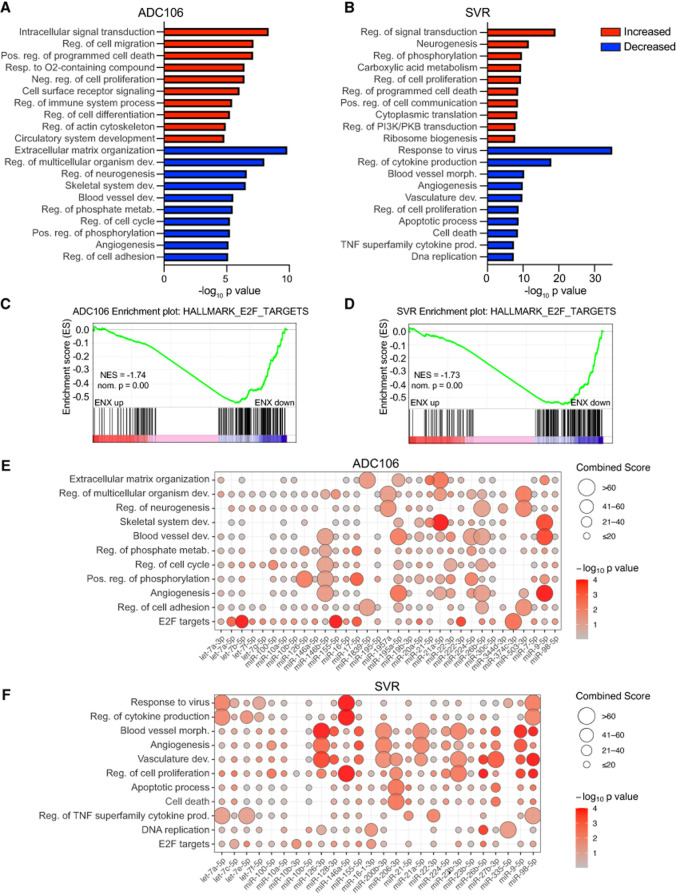
Enoxacin alters mRNA expression. (*A*,*B*) DAVID gene ontology analyses of significantly upregulated (red) and downregulated (blue) genes in ADC106 (*A*) or SVR (*B*) cells. (*C*,*D*) GSEA of the hallmark pathway “E2F targets” in the ADC106 (*C*) and SVR (*D*) cells. Normalized enrichment scores (NESs) and nominal *P*-values are shown. (*E*,*F*) Enrichment plot of potential miRNA-regulated genes, with the downregulated genes (FDR < 0.05, log_2_ fold change <−0.5) within the top oncogenic pathways based on DAVID gene ontology or the overlapping hallmark GSEA terms in ADC106 (*E*) or SVR cells (*F*) treated with 100 µM ENX or DMSO.

To determine whether ENX also influences mRNA expression more broadly, we performed RNA-seq on SVR and ADC106 cells treated with ENX or DMSO for 72 h. We identified 1689 and 3733 differentially expressed mRNAs (absolute log_2_ fold change > 0.5, FDR < 0.05) in ADC106 and SVR cells, respectively, compared to DMSO-treated controls, with a slight majority being downregulated (Supplemental Fig. S4A–C). To gain insight into the biological processes affected by ENX treatment, we next performed gene ontology (GO) analysis using DAVID. GO analysis of the upregulated genes (FDR < 0.05, log_2_ fold change > 0.5) identified terms such as programmed cell death, negative regulation of cell proliferation, cell differentiation, and ribosome biogenesis (Fig. 5A,B; Supplemental Tables S3, S4). Notably, several pro-apoptotic, growth arrest, and differentiation genes such as *Gadd45a*, *Rb1*, *Rassf6*, Wwox, *Dapk1*, *Rasa3*, *Btg3*, *Steap1*, *Klf4*, and *Deptor* were upregulated in both cell lines (Supplemental Fig. S4D), whereas others, such as *Cdkn1a*, *Rb1*, *Fas*, *Rassf5*, *Chd5*, and *Plk3,* were increased in only one of the two. In contrast, among the GO pathways associated with the downregulated genes, we observed common terms such as cell cycle, angiogenesis, and vascular development (Fig. 5A,B; Supplemental Tables S5, S6). Angiogenesis-related genes such as *Angpt1*, *Egfl7*, *Flt4*, *Notch4*, and *Tek* were consistently downregulated in both cell lines (Supplemental Fig. S4E). We also conducted gene set enrichment analysis (GSEA) and evaluated the top five enriched hallmark pathways in both cell lines based on absolute normalized enrichment scores (NESs). The cell cycle–related pathway “E2F Targets” was significantly suppressed in both cell lines (Fig. 5C,D; Supplemental Fig. S5A–C). In SVR cells, pathways involved in cell stress response and inflammation were also suppressed, while IL6–JAK–STAT3 signaling was activated. Interestingly, TNFα signaling, oxidative phosphorylation, and the P53 pathways were enriched in ADC106 cells, whereas E2F targets and G2/M checkpoint pathways were decreased. Collectively, these data indicate that ENX broadly impacts mRNA expression.

### Enhanced miRNA expression regulates oncogenic pathways

We hypothesize that the anticancer properties of ENX can be attributed to the increased abundance of mature tumor-suppressing miRNAs. To test this, we evaluated the enrichment of miRNA-regulated genes within the downregulated DAVID GO and E2F Targets GSEA pathways using miRTarBase for experimentally validated miRNA-target genes (Huang et al. 2020). Indeed, each suppressed pathway contained at least one statistically significant miRNA with enriched target genes (Fig. 5E,F; Supplemental Tables S7, S8). Some pathways, such as the E2F targets, cell proliferation, and angiogenesis pathways, contained multiple miRNAs with enriched target genes. Interestingly, different miRNA target genes were enriched in each cell line. For example, in ADC106 cells, downregulated let-7 target genes were involved in cell cycle progression and E2F targets. In contrast, in SVR cells, let-7 target genes involved in cytokine production were more prominently downregulated. Consistently, miR-9 target genes in both cell lines were associated with angiogenesis, while miR-146 target genes were linked to both cell proliferation and angiogenesis.

**FIGURE 6.**
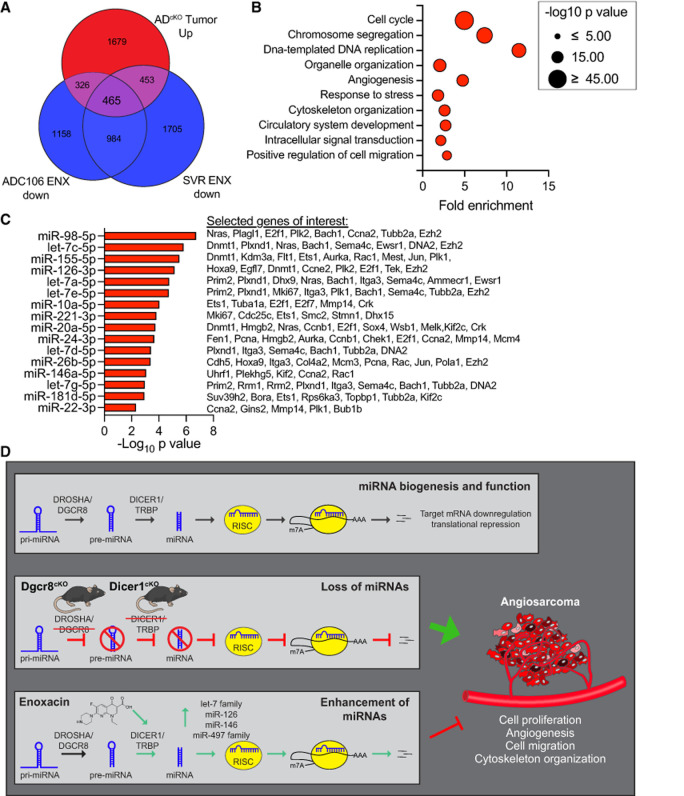
Commonly enriched miRNAs regulate cell cycle, angiogenesis, and cell migration pathways in angiosarcoma. (*A*) Euler diagram of the overlapping genes that are increased in expression in *aP2-Cre;Dicer1*^*Fl/Fl*^ (AD^cKO^, from GSE85834) tumors compared to normal aorta (FDR < 0.05, log_2_ fold change > 0.5) and all genes with decreased expression in ADC106 or SVR cells (FDR < 0.0). (*B*) DAVID gene ontology analysis of enriched terms within the 465 overlapping genes from (*A*). (*C*) The top miRNAs with increased abundance in common between ADC106 and SVR cells treated with ENX with enrichment of target genes among the overlapping genes from (*A*). All miRNAs with significant (FDR < 0.05) enrichment of target genes plotted. Selected oncogenic genes experimentally validated to be regulated by miRNAs are indicated. (*D*) Graphical abstract illustrating normal miRNA biogenesis and function of miRNAs. Conditional *Dgcr8* or *Dicer1* deletion in aP2-Cre expressing cells disrupts canonical miRNA biogenesis, leading to angiosarcoma development in mice. This highlights the important tumor-suppressing role of miRNAs in angiosarcoma. Pharmacologic repurposing of the antibiotic enoxacin enhances the expression of several tumor-suppressing miRNAs and demonstrates anticancer properties in angiosarcoma by repressing oncogenic pathways such as cell proliferation, angiogenesis, cell migration, and cytoskeleton organization. Mouse illustration from NIAID NIH BIOART Source (bioart.niaid.nih.gov/bioart/279).

To identify critical pathways in AS potentially regulated by miRNAs, we determined the intersection between genes downregulated by ENX in both cell lines and the genes upregulated in *Dicer1*^*cKO*^ AS, where miRNA expression is lost (Fig. 6A; Hanna et al. 2017). Gene ontology of this shared gene set revealed enrichment for pathways involved in cell cycle regulation, angiogenesis, and migration (Fig. 6B). Finally, miRTarBase analysis of the 465 genes (limited to just the 50 miRNAs increased in abundance in both cell lines) revealed significant enrichment of well-known tumor-suppressing miRNAs as potential regulators of many of these oncogenic genes including let-7 family (miR-98, let-7c, let-7a, let-7e, let-7d, and let-7g), miR-155-5p, and miR-126a, among others (Fig. 6C). These data suggest that ENX treatment exerts its antitumor effects by restoring miRNA-mediated suppression of oncogenic transcripts and thereby inhibits key pathways such as cell proliferation, angiogenesis, and migration (Fig. 6D).

## DISCUSSION

Most human tumors exhibit a global repression of miRNAs, indicating the importance of downregulating tumor-suppressing miRNAs in cancer (Lu et al. 2005; Merritt et al. 2008; Orellana and Kasinski 2015; Gilles and Slack 2018). In this study, we demonstrated that *Dgcr8* loss in *aP2-Cre* expressing cells phenocopies the AS development driven by *Dicer1* deletion, indicating global miRNA repression as the molecular driver of tumorigenesis. *Dicer1* downregulation promotes tumor development in many mouse models of cancer (Kumar et al. 2009; Lambertz et al. 2010; Llaguno et al. 2023). We have previously shown that *Dicer1* is a classic tumor suppressor in endothelial cells, in which loss of both alleles leads to tumor development (Hanna et al. 2017; Hanna 2022). In contrast, in most other cell types, DICER1 functions as a haploinsufficient tumor suppressor, in which loss of both alleles is detrimental to tumor development and cell survival (Kumar et al. 2009; Lambertz et al. 2010). We find that endothelial cells not only survive *Dicer1* or *Dgcr8* biallelic deletion, but this is transformative and leads to aggressive AS development. This indicates that endothelial cells are uniquely sensitive to miRNA loss–mediated transformation.

Although individual miRNAs may have tumor suppressive or oncogenic functions, global suppression of miRNAs in endothelial cells drives tumorigenesis. This underscores the critical tumor-suppressive role of miRNA regulation of gene expression in AS. Based on our observations of AS development with miRNA loss, we hypothesized that AS cells may be sensitive to miRNA restoration–based therapies. Thus, we tested enoxacin (ENX), a fluoroquinolone antibiotic, previously shown to enhance miRNA biogenesis (Zhang et al. 2008; Melo et al. 2011). ENX is thought to act by enhancing interactions between TRBP and pre-miRNA hairpins, thereby increasing DICER1-mediated processing of the hairpins (Shan et al. 2008; Melo et al. 2011). However, this precise mechanism is still unclear. Genetic studies indicate that DICER1 is required for ENX effects, but some studies call into question the role of TRBP (Abell et al. 2017). Nonetheless, consistent with the tumor-suppressive effects of miRNAs in AS, we found that AS cell lines treated with ENX were less proliferative and migratory and exhibited increased apoptosis.

By small RNA-seq, we determined that ENX induced significant changes in the expression of many miRNAs, while a slight majority of miRNAs were enhanced with ENX in both cell lines, a significant number were also decreased as has been previously reported (Shan et al. 2008; Melo et al. 2011; De Vito et al. 2012; Rocha et al. 2020; Schlösser et al. 2025). Based on published literature and our own data, we found that TARBP2-dependent miRNAs are more likely to be upregulated upon ENX treatment. De Vito et al. identified a group of TARBP2-dependent miRNAs that show pre-miRNA accumulation and reduced mature miRNA levels upon TARBP2 depletion (De Vito et al. 2012). Notably, ENX treatment led to increased mature miRNA expression for these TARBP2-dependent miRNAs. Consistent with their findings, we analyzed these miRNAs in our data set and observed that most of these TARBP2-dependent miRNAs (such as let-7, miR-10, miR-100, miR-125, miR-181, miR-26, and miR-99) were upregulated in both SVR and ADC106 cells after ENX treatment. Additionally, miRNAs that are highly abundant in parental cells seem more likely to benefit from ENX-mediated enhancement of TRBP–Dicer processing, leading to greater maturation efficiency. Using expression levels in DMSO-treated cells, we identified the top 10 most abundant miRNA families and found that eight (SVR) and six (ADC106) of these families were significantly upregulated following ENX treatment. For the downregulated miRNAs, like others have speculated, we hypothesize this could be due to secondary consequences or pleiotropic effects of ENX altering miRNA expression or stability.

We identified several well-characterized tumor-suppressive miRNAs with significantly increased abundance in both ADC106 and SVR cells. Notably, multiple members of the let-7 family were increased. let-7 has widely been implicated as a tumor suppressor in many cancer types and is known to regulate several oncogenes and mediate cell proliferation, differentiation, and apoptosis (Gilles and Slack 2018). miR-126 was also significantly increased with ENX; this endothelial-enriched miRNA has been shown to maintain vascular integrity and inhibit tumor angiogenesis, further supporting its potential as a tumor suppressor in AS. Importantly, we also observe significant increased expression of miR-16-5p and miR-195-5p, members of the miR-15/16/497/424 family, in the ADC106 cells and a subtle increase in miR-16 in the SVR cells. We and others previously identified this family as a potent regulator of cell proliferation, apoptosis, and cell migration in AS (Chen et al. 2016; Benton et al. 2024b). Future studies on the individual and combined functions and mechanisms of these miRNAs will be important.

In addition to its anticancer effects (Melo et al. 2011; Kim et al. 2023), ENX has also been investigated as an antiviral (Schlösser et al. 2025), as a treatment for ALS (Emde et al. 2015; Magen et al. 2025), to prevent obesity and extend life span (Pinto et al. 2018; Rocha et al. 2020), and in the context of inhibiting osteoclasts in bone disease (Toro et al. 2012; Vracar et al. 2018). As an antibiotic, ENX was previously approved by the FDA, but is no longer widely used due to better efficacy with other drugs and potential side effects. However, due to its established clinical history, ENX may have a relatively straightforward path as an anticancer agent, either as a monotherapy or in combination with other therapies. Together, these findings establish the proof of concept for ENX as a therapeutic approach for AS. Future mechanistic and in vivo studies will be essential to determine if the miRNA-restorative and anticancer effects observed in vitro translate into therapeutic benefit in in vivo models of AS.

## MATERIALS AND METHODS

### Mouse studies

All mouse strains were previously generated and described including *aP2-Cre* (*Tg(Fabp4-Cre)1Jmgr*), (MGI: 5300925) (Tang et al. 2008), *Dgcr8*^*Flox*^ (B6n.Cg-Dgcr8tm1.1Blel/Mmjax), (MGI: 4458072). All mice were maintained on a mixed genetic background. Therefore, littermate controls were used for each study. Mice were fed and watered ad libitum in a facility with maintained humidity and ambient temperature with 12 h light–dark cycles. Genomic DNA was isolated from tissue using the DNeasy Blood and Tissue Kit (QIAGEN 69504). *aP2-Cre* and *Dgcr8* genotypes were determined as described previously and primers detailed in Supplemental Table S9 (Tang et al. 2008; Rao et al. 2009).

All experiments involving animal studies were reviewed and approved by the Purdue University Institutional Animal Care and Use Committee (protocol 1908001941).

### Cell culture

Cells were obtained from the following sources: SVR (ATCC CRL-2280, RRID:CVCL_6455), ADC106 (derived from our laboratory as previously described [Hanna 2022]), HTHM (HUVEC cells immortalized with large T [genomic] antigen, hTERT, and MYC) (Searcy et al. 2023), and HEK-293T (RRID:CVCL_0063, M. Kazemian, Purdue University). The SVR, ADC106, and HEK-293T cells were maintained in DMEM (HyClone SH30243) with 10% FBS (HyClone SH30910.03), 1× antibiotic–antimycotic penicillin, streptomycin, and amphotericin B (PSA) (Sigma-Aldrich A5955). The HTHM, KU-CAS3, and KU-CAS5 cells were maintained in VascuLife VEGF endothelial media (Lifeline Cell Technology LM-0002), corresponding supplementary kit (Lifeline Cell Technology LS-1020), and 1× PSA. All cells were incubated at 37°C in 5% CO_2_. Cell line STR profiles are provided in Supplemental Table S10. Cell lines were passaged no more than 15 times past the initial thaw, and mycoplasma testing is performed three times annually, with each experiment being performed within 4 months of the last mycoplasma testing.

Cells were treated with DMSO or enoxacin (ENX) (Thermo Scientific J61912.06). Cell viability was determined using the CellTiter-Glo Assay (Promega G7570), and apoptosis was determined using the Caspase 3/7 Glo Assay (Promega G8090) measuring luminescence with a BioTek Synergy 2 (BioTek/Agilent, RRID:SCR_020536). Population doubling assay was performed as previously described (Hanna et al. 2018). In brief, 50,000 cells were seeded, and every 3 days, cells were lifted and counted using the LUNA-II Automated Cell Counter (Logos Biosystems). Then, 50,000 cells were reseeded for continued culture. Colony formation was performed by seeding 50 or 100 cells per well in 6 well plates and culturing them under standard conditions. When visible colonies had formed, cells were stained with crystal violet for 15 min. Excess stain was rinsed off with distilled water, and plates were air-dried. Transwell assays were performed with transwell permeable supports (Corning 3422). Cells were suspended in serum-free DMEM, and DMEM supplemented with 10% FBS was used as a chemoattractant. Nonmigrated cells were removed from the inner chamber with a cotton swab, and migrated cells were crystal violet stained, imaged, and quantified. At least three independent fields per sample were used for quantification of images. Dual-luciferase assays were performed by seeding HEK-293T cells in 48 well plates as described (Hanna et al. 2016). The following day, cells were treated with DMSO or 100 µM ENX. On the next day, cells were transfected with FuGENE6 (Promega E2691) with 5 ng of psiCHECK2 reporter plasmids (empty or a miR-497 sensor in which the Renilla luciferase 3′UTR was modified to include a perfectly complementary miR-497 site, as described previously [Benton et al. 2024b]). Seventy-two hours posttransfection, cells were lysed in passive lysis buffer, and luciferase activity was measured using the Dual-Luciferase Reporter Assay System (Promega E1910) on a SpectraMax multimode plate reader (Molecular Devices, RRID:SCR_023920). Renilla luciferase activity was normalized to Firefly luciferase activity to control for transfection efficiency and analyzed as relative luciferase activity compared to the DMSO-treated empty control.

### Histology, immunohistochemistry, and immunoblots

Hematoxylin and eosin (H&E) histology and immunohistochemistry (IHC) were performed following standard protocols and antibodies against ERG (Thermo Fisher Scientific MA5-32036), Factor VIII (Abcam ab203590), and PECAM1 (Thermo Fisher Scientific MA5-37858). The antibodies and staining protocols details are provided in Supplemental Table S11. Whole cell lysates from cells were prepared in RIPA buffer. Protein concentrations were determined by the BCA protein Assay Kit (Pierce 23225). Lysates were resolved by SDS-PAGE (Invitrogen/Thermo Fisher Scientific NP0321BOX) and transferred to Immobilon-P Transfer Membrane (Merck Millipore IPVH00005). Blots were probed with indicated antibodies (Supplemental Table S11) and visualized using chemiluminescence Luminol reagent (Santa Cruz Biotechnology SC-2048) and imaged using the Azure c600 imager (Azure Biosystems).

### RNA and gene expression

Total RNA was prepared using the Quick-RNA Miniprep Kit (Zymo Research R1054) according to the manufacturer's instructions. cDNA was generated using High-Capacity RNA to cDNA kit (Applied Biosystems 4387406). Relative expression by qRT-PCR was quantified using the delta-delta CT method normalized to *Gapdh* using primers described in Supplemental Table S9. For miRNA expression, the miRCURY cDNA kit (QIAGEN 339340) was followed with qRT-PCR using LNA miRCURY primers detailed in Supplemental Table S9. miRNA expression was normalized to snRNA U6. RNA-seq was conducted on total RNA from ADC106 or SVR cells treated with DMSO or 100 µM ENX for 72 h. mRNA [poly(A) enrichment] and small RNA library prep and sequencing were performed by Novogene on an Illumina NovaSeq platform. Differential gene and miRNA expression were analyzed as previously described (Wang 2024). In brief, for mRNA-seq, raw paired-end sequencing data were processed and quantified using RSEM (v1.3.1) with default parameters, and Bowtie2 as the aligner (Li and Dewey 2011; Langmead and Salzberg 2012). The Bowtie2 reference index was generated using “rsem-prepare-reference” with the GRCm38 transcriptome and RefSeq gene annotations obtained from the UCSC Genome Browser. Differential gene expression analysis was performed using DESeq2 (v1.42.1) (Love et al. 2014). For the small RNA-seq, raw reads were cleaned (adapter trimming, quality trimming Phred quality score >30) using the Fastp toolkit (version 0.23.2) (Chen et al. 2018). miRNA quantification was performed with QuickMIRSeq tool version 1.0 (Zhao et al. 2017). Filtered counts from QuickMIRSeq were used for differential expression analysis with DESeq2 (v1.42.1). Previously published transcriptomic data from *aP2-Cre;Dicer1*^*Fl/Fl*^ (AD^cKO^) was analyzed from GSE85834 (Hanna et al. 2017). Gene ontology analysis was conducted on the differentially expressed genes using the Database for Annotation, Visualization, and Integrated Discovery (DAVID) (Dennis 2003). Classification of terms was analyzed based on biological process terms (BP_FAT) with the exclusion of redundant and general terms. Gene set enrichment analysis (GSEA) (Mootha et al. 2003; Subramanian et al. 2005) was performed against the hallmarks gene set. Enrichment of experimentally validated miRNA-target gene interactions identified by Enrichr (Xie et al. 2021) and the miRTarBase (Cui et al. 2025) database.

### Statistics

Statistical analyses were performed using Prism Version 9 (Graph Pad Software, Inc.). All results are expressed as the mean ± SD unless stated otherwise. Pairwise comparisons were performed with a two-tailed, unpaired Student's *t*-test. Significance cutoff at *P*-values <0.05 was considered significant.

## DATA DEPOSITION

All relevant data and resources can be found within the article and its Supplemental data. The RNA-sequencing data are deposited in the Gene Expression Omnibus (GEO) under accession numbers GSE298851 and GSE298666.

## SUPPLEMENTAL MATERIAL

Supplemental material is available for this article.

## Figures and Tables

**Bozhi Liu:**
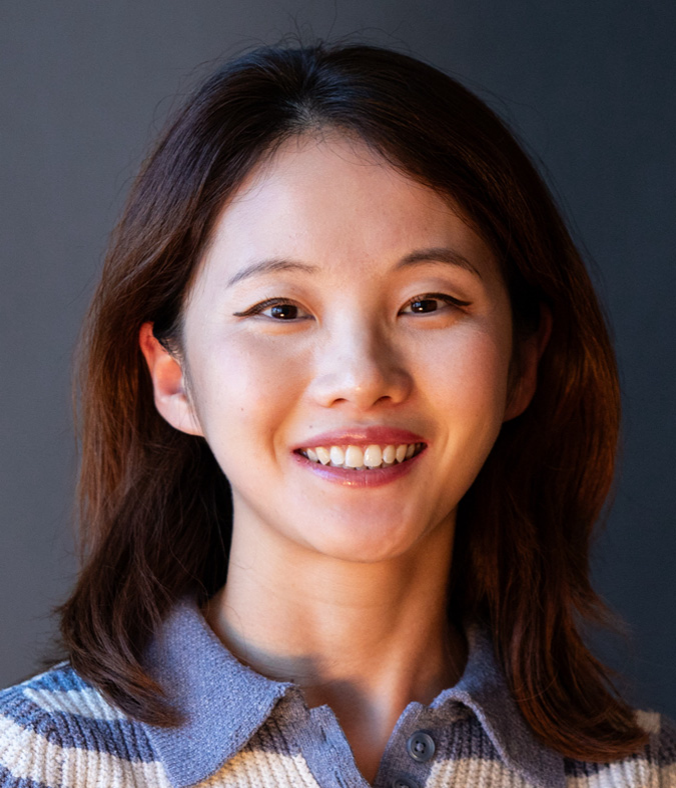


**Annaleigh Benton:**
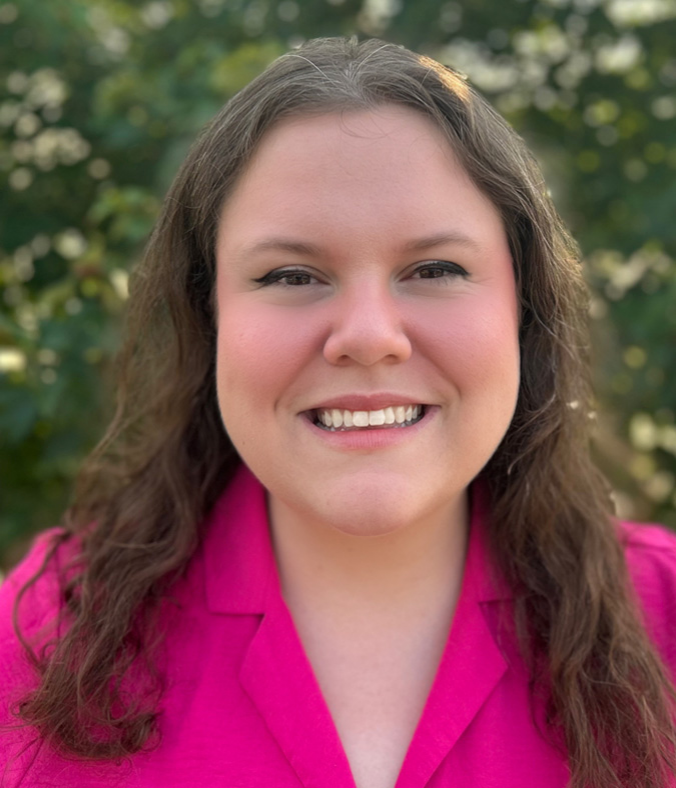


## MEET THE FIRST AUTHORS

***Meet the First Author(s)* is an editorial feature within *RNA*, in which the first author(s) of research-based papers in each issue have the opportunity to introduce themselves and their work to readers of *RNA* and the RNA research community. Bozhi Liu and Annaleigh Benton are co-first authors of this paper, “Canonical microRNA loss drives tumor development, implicating therapeutic efficacy of enoxacin in angiosarcoma,” along with Ant Murphy. Bozhi is a graduate student in Dr. Jason Hanna's laboratory in the Department of Biological Sciences at Purdue University, focusing on CRISPR–Cas9 screening, miRNA-related mechanisms, and model development in angiosarcoma. Annaleigh is a former graduate student in Dr. Jason Hanna's laboratory, where she studied tumor-suppressing microRNAs in angiosarcoma. She is currently an Instructor of Biology at the North Carolina School of Science and Mathematics.**

**What are the major results described in your paper, and how do they impact this branch of the field?**

**BL and AB:** This paper confirms that the global loss of miRNAs contributes to angiosarcoma. We also demonstrated that the miRNA biogenesis enhancer, enoxacin, showed tumor-suppressive effects in angiosarcoma cells and led to the downregulation of oncogenic pathways. Together, these findings establish the essential role of miRNAs in angiosarcoma and highlight the therapeutic potential of miRNA enhancement as a treatment strategy.

**What led you to study RNA or this aspect of RNA science?**

**BL:** In previous studies, we found that the conditional deletion of *Dicer1*, which is an essential gene involved in the miRNA biogenesis pathway, led to angiosarcoma development in a mouse model, indicating that miRNAs contribute to angiosarcoma. To further verify this conclusion and evaluate the therapeutic potential of miRNA enhancement, I pursued this project to investigate the role of miRNAs in cancer biology.

**AB:** I was interested in studying miRNAs in the context of cancer because of their ability to regulate many oncogenic transcripts simultaneously. This aspect of miRNA biology makes them ideal therapeutic candidates, which was exciting to me, and attracted me to the RNA field.

**If you were able to give one piece of advice to your younger self, what would that be?**

**BL:** I would go back and tell the younger myself that “pressure is a privilege—it only comes to those who earned it,” said by a legendary tennis player, Billie Jean King. A PhD journey can be filled with pressure, failure, and moments of frustration. However, I hope my younger self understands that being in a PhD position and carrying the responsibilities of research is also luck. I should be grateful for the opportunity to pursue a PhD degree and to move my projects forward. Throughout my PhD, I never had issues with handling pressure, but understanding this perspective earlier would have allowed me to enjoy my research even more and embrace challenges with greater gratitude.

**AB:** I would advise my younger self that trial and error or “failure” is a natural part of science that is not a personal deficit, but actually very useful in the pursuit of truth. This can be a frustrating part of science, but it's important to stay positive and move forward, because the more information you gather, the closer you get to the answer.

**Are there specific individuals or groups who have influenced your philosophy or approach to science?**

**BL:** First, I want to thank my PI Dr. Jason Hanna, who recognized my potential in my grad school interview, welcomed me into his lab for rotation, and eventually gave me the opportunity to join his lab and work on cancer biology research, which I love most. I really appreciate all his patience, guidance, and encouragement. His door is always open, and he always has time for my questions. With all of this, in the Hanna Lab I gained amazing experience in research and developed strong confidence as a scientist. Second, I want to thank my mentor in the Hanna Lab, Annaleigh Benton, who is also the first author of this paper. I thank her for her patience and time for training me in basic biology skills. She is humorous and definitely has a talent for a talk show. She introduced cancer biology to me in an engaging and funny way. She is professional in mentoring, so I got the chance to learn how to be a good mentor as well. Also, I thank my undergraduate research assistant/mentee, Will, who conducted many qRT-PCRs for this project, and I couldn't have finished this paper without him. All in all, thanks to all the help from my lab mates, and I am very grateful that I am in a lab where I become the best version of myself.

**AB:** My undergraduate professors at the University of North Carolina at Chapel Hill nurtured my early interest in biology and taught me how to approach science methodically. I thank them for their encouragement in my early career, and helping me believe in myself. I also thank my PhD advisor, Dr. Jason Hanna, for his patience and encouragement. He has had the greatest impact on my development as a scientist. He is not only a fantastic scientist, but a great leader of the lab and a genuinely kind person regardless of the situation. I am happy that I have him as an example to look up to when I mentor students in the present and future.

**What are your subsequent near- or long-term career plans?**

**BL:** I want to work in industry and build a reputation with deep expertise in oncology translation research. The motivation started when I worked in clinical research during my master's training. I worked in a hospital, and I saw many patients with advanced hepatocellular carcinoma whose only option was conservative therapy, and it was difficult to see their health decline without effective targeted treatments. This made me want to work on drug development. Also, this experience raised many questions for me: Why are there no effective targeted therapies? What's the driver of cancer progression and metastasis? Then I wanted to understand cancer at a deeper, molecular level. This motivation led me to pursue a PhD degree in cancer biology, where I now work on cancer mechanism studies. I've loved the academic training in my PhD, but in terms of my long-term career goals, I want my work to be more directly connected to drug development and patient impact. Contributing to work that can ultimately benefit patients has been a consistent and powerful motivation for me. I hope that, one day, my discoveries will be translated into therapies that improve patient prognosis.

**AB:** Teaching and mentorship were my favorite aspects of graduate school. Therefore, I chose to pursue a career in education, and I am currently an instructor at the North Carolina School of Science and Mathematics. It is rewarding to work with students with a passion for science.

**How did you decide to work together as co-first authors?**

**BL and AB:** We decided to work together because our projects were all pointing toward the implication of miRNAs in angiosarcoma development. Ant developed the *Dgcr8* knockout mouse model and showed that the deletion of one essential gene in miRNA biogenesis pathway leads to angiosarcoma development in mice. Basilia and Annaleigh were collaborating on the enoxacin project. We used a complementary approach, where we treated angiosarcoma cells with a miRNA enhancer, enoxacin, and found that it has tumor-suppressive effects. Together, these findings highlight that miRNAs contribute to angiosarcoma.
